# Child Homicide Amounting to Murder in Malaysia: Descriptive Analysis of the Statistics and Causes

**DOI:** 10.3389/fpsyg.2022.854539

**Published:** 2022-07-14

**Authors:** Salmi Razali, Nor Hidayah Jaris, Muhamad Zaid Muuti, Nuraisyah Chua Abdullah

**Affiliations:** ^1^Department of Psychiatry, Faculty of Medicine, Universiti Teknologi MARA, Sungai Buloh, Malaysia; ^2^Department of Ethics and Medical Law, Faculty of Medicine, Universiti Teknologi MARA, Sungai Buloh, Malaysia; ^3^Faculty of Law, Universiti Teknologi MARA, Shah Alam, Malaysia; ^4^Institute of Pathology, Laboratory and ForensicMedicine (I-PPerForM), Universiti Teknologi MARA, Sungai Buloh, Malaysia

**Keywords:** child homicide, rates of child homicide, background offenders, Malaysia, motives

## Abstract

**Objective:**

To describe the child homicide rates and examine the circumstances of homicides of children in Malaysia from January 2010 to June 2021.

**Methods:**

This is a retrospective secondary analysis of police records on child homicides in Malaysia. Background demographic characteristics of victims, suspected offenders, motives of homicide, and rates of child homicide in Malaysia were analyzed descriptively.

**Results:**

Three hundred thirty-two child homicide cases involving 349 children aged 18 years old and below by 458 suspected offenders were described. The Malaysian rates of child homicide from 2010 to 2018 fluctuated between 0.28 and 0.37 per 100,000 population of children aged 18 and below. The rates show decreasing trend to 0.19 and 0.17 per 100,000 population of children aged 18 in 2019 and 2020, respectively. Most of the victims were Malay and Indians, boys, aged 0–5 years old and 16–18 years old. Offenders were likely to be someone known to the victims, including parents, stepparents, and friends, and have unstable incomes. The main possible motives of homicide are jealousy and revenge, child abuse, and feeling distress.

**Conclusion:**

The rates of child homicide in Malaysia are lower compared to other countries and show decreasing trend during this COVID-19 pandemic. These findings perhaps are underestimated and should be cautiously interpreted. Nevertheless, the results should inform immediate intervention to target the at-risk groups.

## Introduction

The death of a child, in any circumstances, is tragic. Where a child dies as a result of assault or neglect, the impacts are not only felt by the child’s immediate family and friends but by the entire community. This led to the legal principle that homicide is an offence against the state. This article focuses on child homicide which is within the scope of the criminal offences of murder rather than other crimes that might potentially involve the death of a child victim. In this discussion, “Child” is referred to as a person under the age of 18 years (United Nations Children’s Fund, 2022).

## Literature Review

Child homicide is a form of violent behavior that causes death to any child under 18 years old (Jung et al., 2020). The term often overlaps with other domestic homicides such as filicide (child-killing by parents), infanticide (killing a child aged less than 1 year old), neonaticide (killing a child aged less than 24 h), and familicide (the killing of a spouse and one or more children; Karlsson et al., 2021; Kim and Merlo, 2021).

The (UNODC, 2019) indicated that from 2008 to 2017, a total of 205,153 children aged 0–14 years old had died due to homicide. In 2016, the United States had the highest rate of homicide of 3 per 100,000 population of children aged 0–17, while Europe marked the lowest rate of 1.6 per 100,000 population of children aged 0–17 (UNODC, 2019). For Asia, the report by United Nations Children’s Fund (UNICEF, 2015) indicated that India and Pakistan recorded the highest number of children homicide cases. In the South-East Asian region, Indonesia, Thailand, and Timor-Leste are the three countries with the highest homicide rate of 0.4–2.3 per 100,000 population (World Health Organization, 2021).

Malaysia is a multi-cultural, multi-religious, multi-ethnic country located in South-East Asia. Malaysia’s population is estimated to be at 32.67 million in the third quarter of 2021, and there are 9.13 million children in Malaysia, which is 28% of the total population, with 4.7 million being boys and 4.42 million being girls (Department of Statistics Malaysia, 2022). Child homicide or murder is a crime that is punishable by death under Section 302 of the Malaysian Penal Code (Act 574). The death of a child may also occur when the child is being abandoned (section 317 of Act 574), killing a baby by a mother who suffers postpartum mental health problems (offence of infanticide under section 309A), and child maltreatment. Fortunately, the number of cases of homicide has been steadily declining from over 600 cases in 2006 to over 400 cases in 2017 (Hakim et al., 2019). However, the datasets for homicide involving children below the age of 18 years old are not widely available and often come from anecdotal sources such as newspaper reports.

Various factors have been suggested to be associated with child homicide, including a child, perpetrators, and surrounding proximal (e.g., parents, family, and caregivers) and distal elements (e.g., economic difficulties, social inequalities, legislation, and enforcement). During a 10-year-period study done by the United Nations of Drugs and Crime, 59% of males and 41% of females were found to be the victims of the murder (UNODC, 2019). It was reported a saddle shape distribution of cases, whereby the highest victims are primarily children aged from 15 to 19 years old (57%) and below 5 years old (20%; UNICEF, 2014). The male gender constitutes 70% of the cases worldwide, particularly in late adolescence. However, in the South-East Asian regions, no marked gender differences among the victims of homicide have been documented (World Health Organization, 2021). It has been suggested that children with disability also tend to be the victim of homicide (Debowska et al., 2015).

In terms of the perpetrator, a systematic review of 9,431 studies from 44 countries highlighted that more than half or 56.5%, of child homicides, were filicide cases (child homicide committed by parents; Stöckl et al., 2017). The majority (77.8%) of filicide victims were under 1 year old (Stöckl et al., 2017). Neonaticide and infanticide are often committed by the mother (Pitt and Bale, 1995; Spinelli, 2008; Porter and Gavin, 2010). The killing of the older child, most often by the mother, father, stepparent, friend or stranger (Stöckl et al., 2017; Kim and Merlo, 2021; Michaels and Letson, 2021).

It is suggested that structural disparities, social differences as well as gender inequality contributed to child homicide (Kim and Merlo, 2021; Razali et al., 2021; Oikawa et al., 2022). Perpetrator’s background includes mother’s childhood abuse, victimization experience, and mental illness (Debowska et al., 2015; Razali et al., 2019), and father’s anti-sociality, aggressive behavior, criminal conviction, substance abuse, and mental illness (such as depression, psychosis, dissociative disorder) are contributing to the act of killing (Debowska et al., 2015; Karlsson et al., 2021). Furthermore, earlier landmark studies proposed that child homicide by parents or filicide was motivated by altruistic thoughts, acute psychosis, child maltreatment, unwanted child or newborn, and spouse revenge (Resnick, 1969, 1970). Further studies highlighted mental health problems and psychological background, level of acceptance or denial of the pregnancy, the pattern of parent-infant attachment, and personality (D'Orbán, 1979; Bourget et al., 2007; Barone et al., 2014; Eriksson et al., 2020).

Very few studies have published literature emphasizing child homicide and filicide cases in Malaysia. This study aims to describe the child homicide rates and examine the circumstances of homicides of children in Malaysia from January 2010 to June 2021.

## Materials and Methods

### Data Sources

Child homicide is defined as a case of the killing of a child aged 18 years old and below. In Malaysia, after a case of child homicide is reported, an investigation file is opened. Police and forensic investigations are conducted by the Special Investigation Division (D9) of the Criminal Investigation Department (CID). This division only monitors data on murder under section 302 of the Act 574. The data do not include cases related to infant abandonment, infanticide, and neonaticide, such as cases punishable under the section 309A (infanticide), and section 317 (child abandonment) of Act 574, which are monitored by the Child Unit of the Sexual and Child Investigation Division (D11) of the CID. According to the Explanation of section 317 of the Act 574, if the child dies in consequence of the exposure or abandonment, the perpetrator may be charged with murder under section 300 or culpable homicide under section 299 of the Act 574, depending on the nature and circumstances of the case.

If the child dies as a consequence of the exposure or abandonment, a person can be charged with murder or culpable homicide. However, the challenge is to determine the *mens rea* of causing death at the time of abandoning or exposing the child. The intention or knowledge to cause the death requires the public prosecutor to prove the accused person’s high degree of *mens rea*. By virtue of section 300(d) of the Penal Code, even if the accused has no intention to cause the child’s death, it must be proven that he knows that the act of abandoning or exposing the child is so imminently dangerous that it must in all probability cause death (Law of Malaysia, 2022). Therefore, the accused person cannot simply be convicted for the offence of murder in cases of baby dumping due to the different degrees of *mens rea*. Furthermore, it is hard to prove that the abandonment or exposure death caused the child’s death. As opposed to the offences of murder or culpable homicide, if the prosecutor succeeds to ascertain the intention or knowledge to cause the death of the baby, the punishment will be the death penalty for murder or imprisonment, which may extend to 30 or 10 years for culpable homicide, depending on the degree of probability of causing death.

For this study, communication with the D9 division was made through the public relation unit. The corresponding police officer was assigned to facilitate the retrieval of data on child homicide from 2010 to 2021 June. Moreover, the officer gathered data from the Department of Statistics, Malaysia, on the total population and number of population of children aged 18 and below from 2010 to 2021.

### Data Analyses

Data from 2010 to 2020 were calculated to provide (i) child homicide rate per 100,000 population of children aged 18 and below and (ii) child homicide rate per 1 million population. For the earlier rate, we divided the number of child homicide cases of a victim aged 18 and below by the number of total children population aged 18 years and below and multiplied by 100,000. For the latter, we divided the number of child homicide cases of victims aged 18 and below by the total population and multiplied by 1 million. All data include the number of citizens and non-citizens. We also describe the sociodemographic backgrounds of the victim, the perpetrators, and possible associated motives as recorded in police records.

## Results

### Child Homicide Rates

Table 1 illustrates that from January 2010 to June 2021, a total of 332 cases of child homicide were reported. The rates of child homicide in Malaysia fluctuated between 0.28 and 0.37 per 100,000 population of children aged 18 and below or 0.86–1.16 per 1 million population from 2010 until 2018. The rates show a decreasing trend of 0.19 and 0.17 per 100,000 population of children aged 18 and below, or 0.58 and 0.52 per 1 million population in 2019 and 2020, respectively. Refer to Table 1 for further details.

**Table 1:** Number and estimated rates of child homicide for Malaysia from January 2010 to June 2021.

Year	Number of child homicide case^b^	Children population aged 18 and below^a^ (*0000)	Child homicide rate (per 100,000 children aged 18 and below)	Total population^a^ (*0000)	Child homicide rate (per 1 million population)
2010	33	10,084.4	0.33	28,588.6	1.15
2011	28	10,058.4	0.28	29,061.9	0.96
2012	28	10,038.5	0.28	29,509.3	0.95
2013	38	10,051.0	0.38	30,214.0	1.26
2014	34	10,027.1	0.34	30,708.4	1.11
2015	29	10,024.0	0.29	31,186.4	0.93
2016	37	10,027.1	0.37	31,633.3	1.17
2017	37	10,006.4	0.37	32,022.4	1.16
2018	28	9,985.8	0.28	32,382.3	0.86
2019	19	9,922.5	0.19	32,582.0	0.58
2020	17	9,811.2	0.17	32,675.1	0.52
2021	4 (January-June)	-	-	-	-

All data include number of Malaysian and non-Malaysian.

a Data source from Department of Statistics, Malaysia.

b Data source from Special Investigation Division (D9) of the Criminal Investigation Department (CID), Royal Malaysian Police.

*to multiply by 1,000

### Background of Victims

Table 2 illustrates that from the total 332 cases, a few cases involved more than one victim, and the total number of victims was 349. About two-thirds of cases (*n* = 222; 63.6%) were male victims. Saddle shape age distribution of victims with about a third (*n* = 130; 37.3%) victims aged between the 5 years of life and another third (*n* = 117; 33.5%) victims aged between 16 and 18 years old. Of the total cases, 137 (39.3%) were Malays, followed by Indian (*n* = 98; 28.1%), Chinese (*n* = 54; 15.5%), and other ethnicities or immigrants.

**Table 2:** Background of child homicide victims in Malaysia from January 2010 to June 2021.

Victim	*N* = 349	%
Sex		
Male	222	63.6
Female	127	36.4
Age (year)		
0–5	130	37.3
6–10	48	13.8
11–15	54	15.5
16–18	117	33.5
Ethnic		
Malay	137	39.3
Indian	98	28.0
Chinese	54	15.5
Others	60	17.2

### Background of Offender

About one in five of the suspected offenders (*n* = 97; 21.2%) were young people aged between 10 and 20 years old, the highest group (*n* = 162; 35.4%) was those between 21 and 30 years old, followed by suspected offenders aged 31–40 years old and older. More than two-thirds (*n* = 353; 77.1%) were male. The majority of the alleged offenders were among those without a stable job or unemployed, reflecting their low socioeconomic statuses.

### Possible Motive

The classification of the motive of homicide was pre-prepared in the police records and is illustrated in Table 3. About one-third (*n* = 107; 30.7%) of the cases were revenge of jealousy, followed by child abuse (*n* = 80; 22.9%), distress (*n* = 40; 11.5%), and others (Table 3). About 23.8% of data on motive were unknown.

**Table 3:** Motive of child homicide in Malaysia from January 2010 to June 2021.

Motives	*N* = 349	%
Revenge/jealousy	107	30.7
Abuse	80	22.9
Distress	40	11.5
Neglect	16	4.6
Rape/burglary	15	4.3
Substance/alcohol intoxication	6	1.7
Poisoning	2	0.5
Unknown	83	23.8

## Discussion

The estimated child homicide rates are calculated from January to June 2021. The rates fluctuated from 2010 until 2018 and showed a significant reduction in 2019 until 2020. Malaysian child homicide rates are considerably lower compared to other South-East Asian countries, i.e., European countries and the US. However, the data should be interpreted cautiously because the number of cases analyzed in this discussion is confined to murder cases as provided under section 302 and excluded the potential child death cases under section 309A (infanticide) and section 317 (child abandonment) of Act 574. The exclusion occurs perhaps because of the departmental division between the Special Investigation Division (D9) and the Child Unit of the Sexual and Child Investigation Division (D11) of the CID of the Royal Malaysian Police. Another possibility for data segregation was the difficulties in determining the age of the victim and the cause of death (either product of miscarriage or *janin*, stillbirth or murder of newborn infant) in cases involving newborns. The majority of the fetuses or newborn victims who were discarded in exposed and harsh environments (such as sewage, garbage areas) were likely to be severely decomposed or macerated, making it difficult to ascertain whether it is a homicide case or not. We previously described these challenges while investigating cases of illegal infant abandonment and neonaticide using local data from D11 (Razali et al., 2014). The limitation to murder cases is also in view of the fact mentioned earlier, i.e., in the prosecution for the criminal offence of abandonment of the child, the challenge is to determine the *mens rea* of causing death at the time of abandoning or exposing the child. Similarly, the same challenge also arises in the act of causing death to a newly born child by the mother who gives birth to the child, which is an offence section 309A of the Penal Code (Law of Malaysia, 2022). This provision applies when a mother suffers from post-natal depression, and due to the disturbance of her mind, she acts or omits to do something that causes the child’s death.

It is interesting to highlight that the child homicide rates in Malaysia reduced significantly in 2019 and 2020. Surprisingly, this trend does not correlate with the surge in the number of violence against children, family violence, and child maltreatment cases (Bhatia et al., 2021; Rodriguez et al., 2021). In Malaysia, the Social Welfare Department (2021) report indicated an increase in the number of children who were in need of care and protection during the COVID-19 pandemic. Perhaps, restriction of movement or lockdown during the coronavirus pandemic may conceal the fatal act of abuse and neglect, reducing the chances of the case being disclosed and reported (Garstang et al., 2020). On the other hand, staying together at home may increase bonding between the child and the potential offender. The presence of others at home may offer protection or minimize the opportunity of the potential offender to commit homicide.

This study found the saddle shape distribution of child homicide cases in Malaysia. Majority of cases involved victims aged 0–5 years old and teenagers aged 16–18 years old. Such distribution of cases is in keeping with the pattern of child homicide as found in other countries (UNODC, 2019). We also found a two-times higher percentage of males to be the victims of child homicide. These patterns could be due to predominant cases of fatal child abuse at earlier age (Cheah and Choo, 2016) and the delinquency and violence among adolescents (Rahimi et al., 2020). Similar to other studies, this study also demonstrated the well-known fact that biological parents as the main offender of child homicide (Stöckl et al., 2017; Kim and Merlo, 2021). In early childhood, the mother is more likely to be the offender of neonaticide or infanticide (Razali et al., 2014; Kim and Merlo, 2021). The offender for older children is likely to implicate the mother, father, stepparent, friend, or stranger (Stöckl et al., 2017; Kim and Merlo, 2021; Michaels and Letson, 2021).

About 131 (28.6%) cases can be categorized as filicide, of which 114 (24.9%) cases were perpetrated by a biological parent and 17 (3.7%) by a stepparent. These are significantly lower than the findings by Stöckl et al. (2017), which conclude that child homicides are largely (59.7%) perpetrated by parents in 44 countries. However, these results need to be interpreted cautiously as 126 (27.5%) cases d no clear suspect, and, hence, the relationship between the preparator and the victim remains unknown. Refer to Table 4 for further detail on the background of the suspected offenders.

**Table 4:** Background of child homicide offenders in Malaysia from January 2010 to June 2021.

Suspected offender	*N* = 458	%
Age (year)		
10–20	97	21.2
21–30	162	35.4
31–40	134	29.3
41–60	65	14.2
Sex		
Male	353	77.1
Female	105	22.9
Employment		
Labor/factory worker	146	31.9
Unemployed	103	22.5
Babysitter	18	3.9
Student	26	5.7
Homemaker	21	4.6
Private/public worker	15	3.3
Unknown	29	6.3
Missing data	100	21.8
Relationship with victim		
Biological parent	114	24.9
Friend	63	13.8
Intimate partner	28	6.1
In-law or relative	40	8.7
Stepparents	17	3.7
Babysitter	15	3.3
Neighbor	6	1.3
No relationship	49	10.7
Unknown	126	27.5

It is interesting to highlight that the distribution of cases of child homicide is not in keeping with the large population in Malaysia whereby Indians contribute to 6.8% of all the population (Department of Statistics Malaysia, 2021). Our study demonstrated that 28% of the child homicide cases are from Indian ethnicity, which may be due to psychosocial stress. They are the marginalized minority with less political representation and weak social economically (Annatury et al., 2018).

Revenge and jealousy were described as the main reasons for child homicide. These motives have been described by many researchers, including the landmark study by Resnick (1969). It is hypothesized that the offender may have a deficit in personal perception that explains the behavior of dehumanization (perceiving the child as an object), which easily be the target of rage and anger (Carruthers, 2016). Another crucial motive is fatal child maltreatment resulting from child abuse and neglect (Michaels and Letson, 2021). This finding is supported by several studies, including a local review of autopsy records of fatal child maltreatment in one of the local university hospitals (Murty et al., 2006; Michaels and Letson, 2021). Given almost 4,500–5,500 cases of child maltreatment occur every year in Malaysia (Salleh et al., 2018), improving the readiness to prevent child maltreatment should be the main priority (Mikton et al., 2013). It is interesting to discuss that our data from the police documented one of the motives as “distress,” without detailing neither the specific type of psychological problems nor mental illness (such as depression, schizophrenia, or other disorders). In Malaysia, a previous review of maternal filicide cases in two prominent forensic psychiatric hospitals in the country described the presence of psychiatric diagnoses (such as major depression, schizophrenia, personality disorder, and adjustment disorder) in cases of filicide (Razali et al., 2015). More than two-thirds of the cases and the majority of women were within the 12-month postpartum period (Razali et al., 2015). Family dysfunction, including marital discord, domestic violence, and having spouses who were addicted to alcohol or drugs were among the most common motives of homicide (Razali et al., 2015).

It is a limitation of the current study that this study may not fully reflect the demographics of the general Malaysian population. Our descriptive analysis limits our ability to report associations between the variables. It is suggested that future research should gather raw data from the police department so that inferential statistical analyses can be conducted. Furthermore, inconsistent classification of the variables may occur without standard coding (such as relationships between offender and victim, motives of homicide). The available police records also do not provide comprehensive medical history, forensic reports, psychiatric reports, and court proceedings related to the case, which minimize proper determination of the motive of homicide. Moreover, substantial data were not available or unknown that may have influenced study findings. Lastly, it should be noted that the data were confined to murder cases, and other related child fatalities such as death due to postpartum maternal psychiatric illness and infant abandonment were excluded from the current study. Moreover, events causing the death of children due to indirect abuse (such as dangerous driving, driving without due care and attention, drowning without supervision, and others) were excluded from the study. Some of those cases highlight an essential difference between an accident and a crime, which is outside of the scope of this study. We could not define each of the motive categories as the Royal Malaysian Police classified subjectively without following the common classification of motive by experts on child homicide. This is a limitation in Malaysia as no standard definition of motive is available.

Further research could focus on the discussion pertaining to negligence and simple accident which cause child death. Hence, a higher degree of negligence needs to be established in criminal liability than in civil cases. It can be the direction in further research to achieve a more comprehensive analysis of existing data on child homicide. We recommend considering all types of deaths together when developing a future study on child homicide and prevention strategies.

## Conclusion

The rates of child homicide in Malaysia are lower compared to other countries and show decreasing trend during this COVID-19 pandemic. These findings perhaps are underestimated and should be cautiously interpreted because of the segregation of databases, incomprehensive data, and lack of data on other causes of child death and fatality. The trend of reduction in rates during the pandemic contradicting other reports should be explored thoroughly.

## Data Availability Statement

The original contributions presented in the study are included in the article further inquiries can be directed to the corresponding author.

## Author Contributions

SR is the main writer and communicates with the Royal Malaysian Police and Department of Statistics Malaysia for data retrieval and writing. SR, NJ, MM, and NA contributed to the report and review the manuscript. NJ is responsible for all the communication of the publication. All authors contributed to the article and approved the submitted version.

## Funding

This study is funded by the Universiti Teknologi MARA [grant reference number: 600-RMC/GPK 5/3 (233/2020)].

## Conflict of Interest

The authors declare that the research was conducted in the absence of any commercial or financial relationships that could be construed as a potential conflict of interest.

## Publisher’s Note

All claims expressed in this article are solely those of the authors and do not necessarily represent those of their affiliated organizations, or those of the publisher, the editors and the reviewers. Any product that may be evaluated in this article, or claim that may be made by its manufacturer, is not guaranteed or endorsed by the publisher.
